# Generation of Enucleated Erythrocytes From Lin^−^CD45^−^CD133^+^ Cells Isolated From Human Umbilical Cord Blood In Vitro

**DOI:** 10.1155/sci/7714753

**Published:** 2025-11-19

**Authors:** Ji He, Fang Wang, Qigang Zhan, Qi Sheng, Yanling Ying, Wei Zhang, Jinhui Liu, Faming Zhu

**Affiliations:** ^1^Blood Center of Zhejiang Province, Hangzhou 310052, China; ^2^Umbilical Cord Blood Bank of Zhejiang Province, Hangzhou 311100, China

## Abstract

**Background:**

At present, healthcare facilities often face blood shortages because of the low supply of donated blood relative to the high demand. Therefore, efforts to develop red blood cell (RBC) production methods have gained traction. In this work, Lin^−^CD45^−^CD133^+^ cells were isolated from human umbilical cord blood (UCB) and subsequently differentiated into erythrocytes in vitro in serum-free culture medium.

**Methods:**

Lin^−^CD45^−^CD133^+^ cells were prepared from mononuclear cells (MNCs) using magnetic-activated cell sorting (MACS). The characteristics of Lin^−^CD45^−^CD133^+^ cells were confirmed using flow cytometry analysis, colony-forming unit (CFU) assays, morphological analysis, immunocytochemistry (ICC) analysis, and real-time fluorescent quantitative polymerase chain reaction (RT–PCR). Erythrocytes were differentiated in serum-free medium supplemented with stem cell factor (SCF), interleukin-3 (IL-3), erythropoietin (EPO), and FK506 for 13 days, after which autoplasma derived from UCB was added at a concentration of 5% beginning on day 14. Erythroid differentiation and maturation were examined using electron microscopy and flow cytometric analysis.

**Results:**

Lin^−^CD45^−^CD133^+^ cells were successfully obtained from UCB. These cells were slightly smaller than normal RBCs and had a high nucleus-to-cytoplasm ratio. Oct-4 and Nanog were expressed at both the mRNA and protein levels in Lin^−^CD45^−^CD133^+^ cells. Most of the colonies were burst-forming unit-erythroid (BFU-E). After 7 days of in vitro culture, the Lin^−^CD45^−^CD133^+^ cells were negative for CD133 expression and positive for CD45 expression. The percentage of CD71^+^ cells gradually increased, peaked on day 10, and then started decreasing on day 13. The percentage of CD235a^+^ cells increased gradually after day 7 and peaked on day 13. CD240 expression was detected on day 18, with the highest level detected on day 20. The number of erythroid cells increased persistently during differentiation, and their morphology was consistent with that of normal erythrocytes.

**Conclusion:**

An ex vivo culture system was developed that can generate human erythrocytes from Lin^−^CD45^−^CD133^+^ cells isolated from human UCB.

## 1. Introduction

Red blood cell (RBC) transfusion is a procedure for delivering blood to increase the oxygen-carrying capacity of the blood or to treat severe anemia caused by blood disorders [1]. Despite the high demand, there is a shortage due to the low supply from blood donors [2, 3]. This question has sparked interest in the production of functional RBCs in vitro [4, 5]. To date, RBCs have been generated from a variety of cell sources, including somatic stem cells, human embryonic stem cells (ESCs), and induced pluripotent stem cells (iPSCs). All of these methods can generate RBCs with the same hemoglobin (Hb) content, morphology, and biological function as natural RBCs [6–9].

In 2006, the Kucia group [10] (University of Louisville, Kentucky, USA) first isolated pluripotent stem cells (PSCs) with biological characteristics similar to those of ESCs from mouse bone marrow and named them very small embryonic-like stem cells (VSELs). Later, Kucia et al. [11] and Halasa et al. [12] used the same method to isolate cell populations similar to those of rat VSELs in fresh human umbilical cord blood (UCB) using fluorescence-activated cell sorting (FACS) to obtain CXCR4^+^CD133^+^CD45^−^ cells. Several research groups subsequently reported the isolation of three subtypes of VSELs from human cord blood [13–15]: CXCR4^+^lin^−^CD45^−^, Lin^−^CD45^−^CD34^+^, and Lin^−^CD45^−^CD133^+^. Observation and analysis using transmission electron microscopy revealed that Lin^−^CD45^−^CD133^+^ cells derived from human UCB presented typical characteristics of ESCs [16–18]. Lin^−^CD45^−^CD133^+^ cells can express the embryonic transcription factors Oct-4 and Nanog and exhibit unlimited proliferation, self-renewal, and multidirectional differentiation of ESCs [19, 20]. Lin^−^CD45^−^CD133^+^ cells, which can be induced to differentiate into endoderm, mesoderm, and ectoderm cells in vitro, have strong potential differentiation ability and have attracted attention in stem cell research because they can help avoid ethical concerns and certain potential defects associated with ESCs [21, 22]. If used as seed cells for tissue engineering and clinical therapy, these cells have good application prospects [23, 24]. Owing to their ESC-like properties, Lin^−^CD45^−^CD133^+^ cells can theoretically differentiate into various cells. Therefore, Lin^−^CD45^−^CD133^+^ cells represent a new source for obtaining erythrocytes upon being directionally induced to form erythrocytes in vitro. The induced differentiation of Lin^−^CD45^−^CD133^+^ cells into RBCs and platelets in vitro has not been studied. However, owing to their stem cell-like characteristics, in theory, the culture system of cord blood CD34^+^ cells should be suitable for targeted expansion. However, this remains to be investigated.

In this work, Lin^−^CD45^−^CD133^+^ cells were isolated from human UCB via a magnetic-activated cell sorting (MACS) system. The characteristics of the cells were then analyzed, followed by the expansion and differentiation of Lin^−^CD45^−^CD133^+^ cells into erythrocytes in vitro. Finally, a culture system of directed induction and amplification in erythrocytes was established.

## 2. Materials and Methods

### 2.1. Sample Collection and Processing

Human UCBs (*n* = 3) were collected from different pregnant women who delivered healthy full-term pregnancies, with written informed consent obtained from the parents, following the guidelines approved by the ethical committee. Fresh UCB was analyzed within 48 h after acquisition. The study was approved by the Ethical Review Committee of the Blood Center of Zhejiang Province, China.

### 2.2. Mononuclear Cell (MNC) Enrichment in UCB

The collected UCB was diluted 1:1 in phosphate-buffered saline (PBS) and carefully overlaid at a 1:2 ratio onto Ficoll–Hypaque solution (1.077 g/mL) (GE Healthcare, Uppsala, Sweden), followed by centrifugation at 1500 rpm for 35 min. After centrifugation, the buffy coat layer was collected in separate tubes. The layer was subsequently diluted with three times the volume of PBS and centrifuged for 10 min. The pellet was collected and resuspended in PBS to make a single-cell suspension.

### 2.3. Lin^−^CD45^−^CD133^+^ Cell Sorting

A magnetic bead sorting system was used to select Lin-negative cells from the MNCs enriched in UCB with a Lineage Cell Depletion Kit, after which CD45 microbeads were used to select CD45-negative cells. After sorting with a CD133 MicroBead Kit, Lin^−^CD45^−^CD133^+^ cells were obtained. All the microbeads were purchased from Miltenyi Biotec (Bergisch Gladbach, Germany). The procedure was performed according to the manufacturer's instructions.

### 2.4. Flow Cytometric Analysis

The sorted cells were characterized by flow cytometry. Directly conjugated antibodies against CD45-PerCP-cy5.5 (BD Biosciences, Oxford, UK) and CD133-PE (Miltenyi Biotec) were used. The cultured cells were labeled with anti-CD235a-PE (glycophorin A), anti-CD71-FITC, and anti-CD240-PE (BD Biosciences, Oxford, UK) for phenotyping. A FACSCalibur flow cytometer with Cell Quest software (BD Biosciences, Oxford, UK) was used to analyze the cells.

### 2.5. Immunocytochemistry (ICC)

The cells were first treated with 4% paraformaldehyde at room temperature for 10–15 min. Afterward, they were washed with PBS three times before being treated with 0.3% Triton X-100 (Biosharp, Beijing, China) at room temperature for 15 min. Afterward, they were blocked with 5% bovine serum albumin (BSA) for 30 min and subsequently washed with PBS once. Rabbit anti-human Oct-4 and Nanog (Cell Signaling Technology, Boston, USA) antibodies were added as primary antibodies. The cells were incubated overnight at 4°C and then were washed with PBS three times. Sheep anti-rabbit IgG–FITC (Lianke, Hangzhou, China) was added as a secondary antibody. Then, the cells were incubated at 37°C for 1 h and then washed with PBS three times. The cells were then restained with DAPI (Molecular Probes, California, USA) for 5 min and washed with PBS three times. A fluorescence microscope (NIKON TE-2000U; Tokyo, Japan) was used to observe the cells and obtain images.

### 2.6. Real-Time Fluorescent Quantitative Polymerase Chain Reaction (RT–PCR) Analysis

Total RNA was isolated from Lin^−^CD45^−^CD133^+^ cells using an RNeasy Mini Kit (Qiagen, Hilden, Germany) following the manufacturer's instructions. The extracted RNA was treated with ribonuclease-free deoxyribonuclease to remove any contaminating genomic DNA. Then, 2 μg of total RNA was reverse transcribed using the Super Script TM III First-Strand Synthesis System (Invitrogen, Carlsbad, USA) following the manufacturer's instructions to synthesize cDNA.

The expression of the pluripotency markers Oct-4 and Nanog was studied with RT–PCR using a Chromo 4 real-time PCR system (Bio-Rad, Hercules, USA). The amplification conditions for Oct-4 and Nanog were as follows: initial denaturation at 95°C for 5 min, followed by 40 cycles of denaturation at 95°C for 15 s and primer annealing at 60°C for 1 min. The threshold cycle (Ct) was subsequently determined using Opticon Monitor software (Bio-Rad, Hercules, USA). Glyceraldehyde 3-phosphate dehydrogenase (GAPDH) was used as a housekeeping gene. The designed oligonucleotide sequences of the primers are listed in Table 1.

### 2.7. Defibrination of Cord Blood Plasma

The cord blood was centrifuged at 4000 rpm to obtain low platelet plasma. Thrombin was added to the plasma, which was then incubated at 37°C for 1 h, followed by incubation at 4°C overnight. Afterward, the fibrin clots were removed by centrifugation at 1500 rpm and stored at − 20°C until use.

### 2.8. Erythroid Differentiation and Maturation

Stage 1 (days 0–13): Lin^−^CD45^−^CD133^+^ cells were inoculated into serum-free medium (StemSpan) (STEMCELL Technologies, Vancouver, Canada) at a concentration of 10^4^ cells/mL, with 1 ng/mL cytokine FK506 (Sigma–Aldrich, Gillingham, UK), 100 ng/mL stem cell factor (SCF) (Peprotech, EC Ltd, London, UK), 5 ng/mL interleukin-3 (IL-3) (Peprotech), and 3 ng/mL erythropoietin (EPO) (Peprotech). On day 4, the cell culture was diluted 4-fold using the same medium. On day 8, the cell concentration was adjusted to 10^5^ cells/mL, and the cytokines SCF (50 ng/mL), IL-3 (10 ng/mL), and EPO (3 ng/mL) were added.

Stage 2 (days 14–17): Cells at a concentration of 5 × 10^5^–1 × 10^6^ cells/mL were inoculated into serum-free medium. The cytokines SCF (50 ng/mL), EPO (2 IU/mL), and 5% defibrinated autologous cord blood plasma were added.

Stage 3 (days 18–23): No cytokines were added to the serum-free medium, and only 5% defibrinated autologous cord blood plasma was added.

All the cultures were maintained at 37°C in a humidified atmosphere of 5% CO_2_, and all the cells were inoculated in 25 cm^2^ cell culture flasks.

### 2.9. Colony-Forming Unit (CFU) Analysis

A total of 1 × 10^4^ Lin^−^CD45^−^CD133^+^ cells were cultured in serum-free erythroid differentiation medium for 1, 4, 7, or 10 days and then cultured in Methocult 4434 (STEMCELL Technologies, Vancouver, Canada) for 14 days. The formation of the following was observed under an optical microscope (Nikon, Tokyo, Japan): CFU, burst-forming unit-erythroid (BFU-E), CFU-granulocyte, and macrophages (CFU-GM).

### 2.10. Wright Staining

To analyze Lin^−^CD45^−^CD133^+^ cell morphology, the sorted Lin^−^CD45^−^CD133^+^ cells were mixed with normal RBCs and stained with Wright's staining solution. The sizes of the two groups of cells were compared under an optical microscope (Olympus, Tokyo, Japan). The cultured cells were stained for 30 s with Wright's staining solution, mixed with buffer solution for 1 min, and then air-dried and observed under a microscope.

### 2.11. Scanning Electron Microscopy

Electrophotography of erythroid cells cultured from Lin^−^CD45^−^CD133^+^ cells was performed on day 23 using a field emission-scanning electron microscope (Hitachi S-800, Tokyo, Japan). Images were recorded with a scanning microscope image analysis system (ESCAN-4000, Tokyo, Japan).

### 2.12. Detection of Erythrocyte 2,3-Diphosphoglycerate (2,3-DPG)

Erythrocyte 2,3-DPG was detected by enzyme-linked immunosorbent assay (ELISA) according to the instructions of the kit (St. Louis, Missouri, USA).

The cell suspension was diluted in 1 mL of PBS and frozen at − 20°C for 20 min. It was removed and thawed and centrifuged at 2000 rpm for 20 min, after which the supernatant was taken as a sample. A standard solution was prepared. Blank wells, standard solution wells, and test sample wells were set on the enzyme-labeled coated plate. A volume of 50 μL of sample was added to the bottom of each well. After incubation at 37°C for 30 min, the plate was washed, and the enzyme was added for 30 min. The plate was washed again. Color development was performed, and stop solution was added. A regression curve was drawn according to the OD value of the standard solution, and the curve equation was obtained. The OD value of the test sample was substituted into the equation to obtain the corresponding concentration, which was then multiplied by the dilution factor to obtain the intracellular 2,3-DPG concentration of the test sample.

### 2.13. Detection of Erythrocyte ATP

ATP was detected using chemiluminescence, and the operation was performed according to the manufacturer's (Promega, Madison, Wisconsin, USA) instructions.

The cell suspension was diluted with PBS to 1 × 10^7^/mL. Standard solutions were prepared, and blank wells, standard solution wells, and test sample wells were established. A volume of 100 μL of sample was added, with duplicate wells set for each sample. Then, 100 μL of CellTiter-Glo reagent was added to each well, and the mixture was incubated at room temperature for 10 min to stabilize the fluorescence signal. The luminescence value was recorded using a luminescence detector. A regression curve was constructed on the basis of the luminescence values of the standard solutions, and the curve equation was obtained. The luminescence value of the test sample was substituted into the equation to obtain the intracellular ATP concentration of the test sample.

### 2.14. RBC Count

The number of RBCs was detected using a Sysmex XS-900i hematology analyzer (Sysmex Corporation, Kobe, Japan).

## 3. Results

### 3.1. Characteristics of Lin^−^CD45^−^CD133^+^ Cells

**Figure 1:**
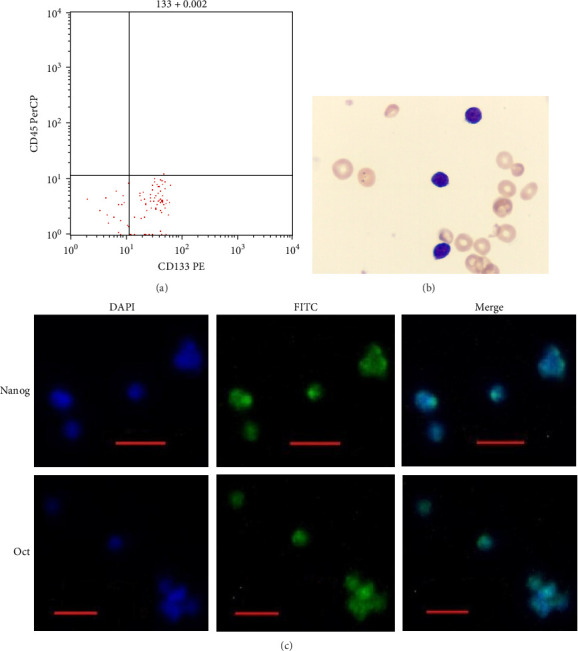
Characteristics of Lin^−^CD45^−^CD133^+^ cells. (A) Flow cytometry results for the sorted cells. (B) Morphology of Lin^−^CD45^−^CD133^+^ cells compared with that of normal RBCs using Wright staining. (C) Expression of Oct-4 and Nanog detected by immunofluorescence (scale bar = 2 μm).

The median initial cord blood volume was 80 mL. After sorting, Lin^−^CD45^−^CD133^+^ cells were obtained from UCB (Figure 1A), and the median number of Lin^−^CD45^−^CD133^+^ cells was 6 × 10^5^. These cells were slightly smaller than normal RBCs. These cells also had a high nucleus-to-cytoplasm ratio (Figure 1B). Oct-4 and Nanog were expressed in Lin^−^CD45^−^CD133^+^ cells according to immunocytochemical results (Figure 1C). Oct-4 and Nanog mRNA was detected in Lin^−^CD45^−^CD133^+^ cells using RT–PCR.

### 3.2. Colony Assay

**Figure 2:**
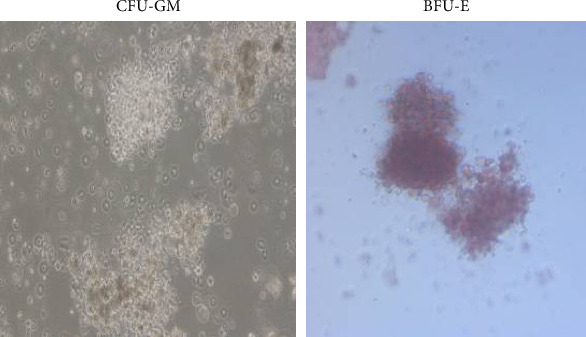
Representative images of the colony-forming unit (CFU) assay for Lin^−^CD45^−^CD133^+^ cells.

A small amount of CFU-GM was detected in Lin^−^CD45^−^CD133^+^ cells cultured for 1 day. CFU-GM was detected at higher levels in cells cultured for 4 days. CFU-GM and BFU-E were present in cells cultured for 7 days, and BFU-E was dominant in cells cultured for 10 days (Figure 2). Therefore, VSEL can differentiate into red erythrocytes only after a period of induction in serum-free erythroid differentiation medium.

### 3.3. Flow Cytometry Analysis of Erythrocytes Differentiated From Lin^−^CD45^−^CD133^+^ Cells In Vitro

**Figure 3:**
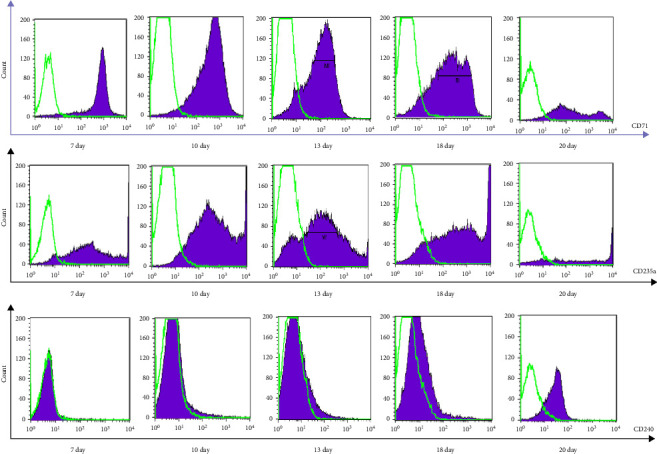
Changes in CD71/CD235a/CD240 expression during erythrocyte differentiation from Lin^−^CD45^−^CD133^+^ cells in vitro.

Erythroid cells were generated from Lin^−^CD45^−^CD133^+^ cells in vitro. After 7 days of culture, the expression of the stem cell surface markers CD34 and CD133 was not detected, whereas positive CD45 expression was detected. The percentage of CD71^+^ cells started to gradually increase on day 1, peaked on day 10, and then started to decrease on day 13. The percentage of CD235a^+^ cells started to increase on day 7 but peaked on day 13. CD240^+^ cells began to shift to the right on day 18, with the highest positive rate occurring on day 20, as shown in Figure 3 and Table 2.

### 3.4. Wright Staining for Erythrocytes Differentiated From Lin^−^CD45^−^CD133^+^ Cells In Vitro

**Figure 4:**
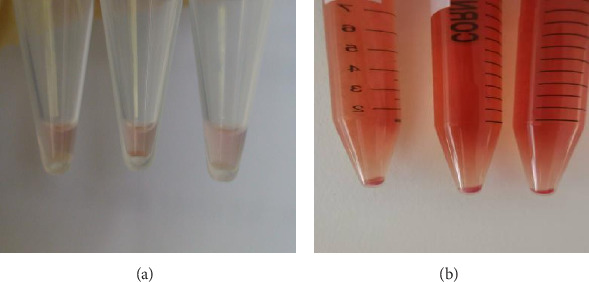
Representative image of red blood cell pellets observed on day 13 (a) and day 23 (b) of culture after centrifugation.

**Figure 5:**
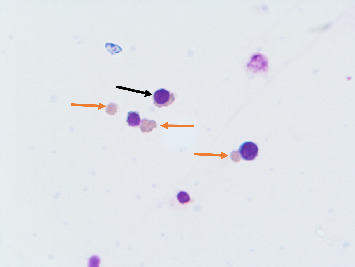
Cell morphology on day 18 of culture. The black arrow shows the orthochromatic normoblast. The red arrow shows enucleated erythrocytes (magnification: 100 ×).

After 23 days of culture, more enucleated erythrocytes were observed, and the centrifuged cells showed red cell packing (Figure 4). Wright staining revealed the morphology of the cultured orthochromatic normoblasts and enucleated erythrocytes (Figure 5).

### 3.5. Scanning Electron Microscopy of Erythrocytes Differentiated From Lin^−^CD45^−^CD133^+^ Cells In Vitro

**Figure 6:**
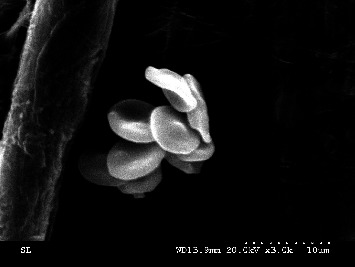
Scanning electron microscopy images of erythroid cells on day 23 (magnification: 3000 ×).

The cells were further studied using electron microscopy for morphological analysis, and biconcave disk-shaped cells were observed (Figure 6).

### 3.6. In Vitro Production of Erythroid Cells

A total of three induction cultures were carried out. The enriched Lin^−^CD45^−^CD133^+^ cells were cultured for 21 days. On the 15^th^ day of culture, many RBCs began to be generated, with a total cell number of 2 × 10^8^.

### 3.7. RBC Function Detection

After 21 days of culture, the contents of ATP and 2,3-DPG did not significantly differ between RBCs and normal RBCs (*p*  > 0.05), as shown in Table 3.

## 4. Discussion

In recent years, VSELs have been considered the holy grail of regenerative medicine to win the three-front war on tissue damage, cancer, and aging [25–28]. VSELs were first obtained by a combination of chemotactic isolation and FACS [14]. First, MNCs were isolated. After the RBCs were removed, SDF-1 was added for chemotaxis. The cells expressing the CXCR4 receptor could react with SDF-1 and be preliminarily isolated. The selected CXCR4^+^ cells were subsequently sorted with a CD45 flag using FACS. Later, a new method combining density gradient centrifugation and FACS was developed [25, 26]. These methods all require FACS technology. However, FACS technology requires a special sorting-type flow cytometer, which is complicated to operate and requires special training and experience for operators to perform flow sorting well. General laboratories cannot meet this requirement. In this study, VSELs (Lin^−^CD45^−^CD133^+^ cells) were obtained using magnetic sorting. The characteristics of Lin^−^CD45^−^CD133^+^ cells were confirmed using flow cytometry analysis, a CFU assay, and morphological analysis. Immunofluorescence and RT–PCR confirmed that the cells expressed the ESC markers Oct-4 and Nanog, indicating that the VSEL cells could be successfully sorted using a magnetic sorting method. This method has the following advantages. First, the equipment required for magnetic sorting as well as the procedure is relatively simple, with less stringent technical requirements for the operators, such that the method can be performed in general laboratories. Moreover, magnetic sorting only places the cell in a weak magnetic field with a negligible impact on the cell.

Bujko et al. [22] evaluated numerous Lin^−^CD45^−^CD34^+^ and Lin^−^CD45^−^CD133^+^ UCB-derived VSELs and reported that the Lin^−^CD45^−^CD133^+^ population was rarer and expressed higher mRNA levels of the pluripotency markers Oct-4 and Nanog. However, the expression of proteins associated with the main biological processes did not significantly differ between the populations of the two cell types. Therefore, Lin^−^CD45^−^CD133^+^ UCB-derived VSELs were selected for in vitro differentiation experiments in this study.

To date, many studies on the generation of RBCs from various sources have been reported [5, 29], including ESCs, iPSCs, and hematopoietic stem cells. Most related studies have focused on the differentiation of hematopoietic stem cells to RBCs in vitro. However, studies on the differentiation of VSELs in vitro remain controversial. Using immunofluorescence detection of specific proteins and detection of cell marker mRNAs, Kucia et al. [11] reported that VSELs can be directly induced to differentiate into cardiomyocytes (mesoderm), neurons, astrocytes, oligodendrocytes (ectoderm), and islet cells. McGuckin et al. [23] also studied the differentiation of VSELs into nerve cells in vitro. McGuckin et al. successfully differentiated nerve cells in vitro using VSELs derived from human UCB. Danova-Alt et al. [30] applied various culture media to differentiate VSELs, including basic culture medium, commercial human hematopoietic stem cell medium, human mesenchymal stem cell medium, human pluripotent stem cell medium, and mouse pluripotent stem cell medium. The results showed that these media did not support human UCB VSEL proliferation or differentiation in vitro. These authors suggested that VSELs derived from human UCB had abnormal karyotypes and did not possess the characteristics of stem cells such that they could not multiply and differentiate [31–33]. Therefore, the induced differentiation of VSELs in vitro still needs further investigation. For the first time, Gounari et al. [34] demonstrated that VSELs can differentiate toward HSCs capable of inducing in vitro hematologic reconstitution in the presence of Wharton's jelly-derived mesenchymal stromal cells. However, erythrocyte generation induced by VSELs in vitro has not been reported.

Previous studies have shown that a serum-free liquid culture system can be used to expand many human UCB CD34^+^ cells and that cultured hematopoietic cells can completely differentiate into RBCs. Therefore, it is possible that one unit of UCB can expand RBCs to meet clinical treatment requirements [8]. A serum-free culture system with cytokines used to study the differentiation of erythrocytes in human UCB CD34^+^ cells has become a mature culture system in our laboratory. The system was established by combining the cytokines EPO, SCF, and IL-3. Erythroid lineage development requires a delicate balance between the opposing effects of proliferation-promoting factors and differentiation-inducing factors. The two most important cytokines are EPO and SCF. EPO protects erythroid progenitor cells from apoptosis by activating antiapoptotic proteins, and it also stimulates Hb synthesis and is essential for terminal differentiation. SCF acts synergistically with this lineage-specific factor by promoting erythroid progenitor cell proliferation. IL-3 promotes mainly cell proliferation and differentiation in the early stage of cell culture. FK506 selectively promoted the proliferation of BFU-E cells. After 20 days of culture, the cell count reached 10^8^. Polychromatic and orthochromatic erythroblasts started to appear on the 15^th^ day of culture, and mature anucleated RBCs appeared on the 18^th^ day. On the 20^th^ day of culture, ATP and 2,3-DPG levels in RBCs were consistent with those in normal RBCs. On this basis, the culture of Lin^−^CD45^−^CD133^+^ cells produced 2 × 10^8^ RBCs, which was similar to the number of RBCs generated by cord blood-derived CD34^+^ cells in our culture system; however, the yield was not as high as that from the iPs or erythroid line [35]. CD71, CD235a, and CD240 antigen-positive rates changed with cultivation time, which indicated that such a culture system can generate human erythrocytes from Lin^−^CD45^−^CD133^+^ cells. Baek et al. [14] reported that the addition of autologous plasma to a serum-free culture system could greatly improve the denucleation efficiency of RBCs, which led to the generation of many nonnucleated and mature RBCs. From day 14 in this study, the cells were cultured in serum medium supplemented with 5% CB. On the 15^th^ day of culture, many orthochromatic erythrocytes and some erythrocytes were observed. The cultured cells appeared as red pellets, indicating that autologous plasma can improve the denucleation efficiency of RBCs in vitro.

To our knowledge, this is the first study on the generation of enucleated erythrocytes from Lin^−^CD45^−^CD133^+^ cells isolated from human UCB in vitro. However, this study has some limitations. First, only the results of three experiments are reported, with a small sample size and limited number of experiments. Second, only the 2,3-DPG and ATP levels of the cultured RBCs were detected, while the oxygen-carrying capacity and lifespan of the cultured RBCs were not measured. Finally, there is a lack of control groups such as UCB-derived Lin^−^CD45^−^CD133^+^ cells and CD34^+^ cells are simultaneously induced and differentiated in vitro to generate RBCs. Now inefficient differentiation is a common problem in the induction of stem cells to differentiate into erythroid cells in vitro. Even if stem cells are successfully differentiated into erythrocytes, the functional maturity of these cells is often insufficient for clinical applications. There are still obstacles to overcome in the large-scale in vitro production of RBCs, and the culture medium and cytokines used in the culture process are relatively expensive. Some refinements have the potential to further expand the culture for the large-scale production of RBC products. In particular, if large-capacity bioreactors can be used to produce RBCs, the cost of in vitro blood cell culture will be significantly reduced. Because Lin^−^CD45^−^CD133^+^ cells are derived from UCB, their safety profiles are better than those associated with the use of genetically modified cells such as iPs and immortalized lines. Given that RBCs are produced in an in vitro environment, they need to be thoroughly characterized. In conclusion, the in vitro culture system for erythroid differentiation by UCB-derived Lin^−^CD45^−^CD133^+^ cells was established in this study, which lays a foundation for the basic research on erythrocyte development and the future production of RBCs suitable for transfusion.

## Figures and Tables

**Table 1:** Primers used for qPCR.

Primers	Sequence
Oct-4	F: TTGATCGCTTGCCCTTCTG
	R: ATCAGCCACATCGCCCA
	P: FAM-CCACACTCGGACCACATCCTTCTCG-TAMRA

Nanog	F: AGATGCCTCACACGGAGACTG
	R: TTTTTGCGACACTCTTCTCTGC
	P: FAM-CCAGTCCCAAAGGCAAACAACCCA-TAMRA

GAPDH	F: GCCAGCCGAGCCACAT
	R: CTTTACCAGAGTTAAAAGCAGCCC
	P: HEX-CCAAATCCGTTGACTCCGACCTTCA-TAMRA

**Table 2:** Flow cytometry analysis of cell surface markers (median).

Marker	7 day (%)	10 day (%)	13 day (%)	18 day (%)	20 day (%)
CD71^+^	89.37	97.95	85.71	83.17	82.35
CD235a^+^	73.39	85.26	95.12	91.56	89.73
CD240^+^	0.65	8.97	10.23	35.67	82.08

**Table 3:** Red blood cell function detection.

Items	Cultured red blood cells	Normal red blood cells
ATP (umol/L)	1.946 ± 3.16	2.143 ± 2.85
2, 3-DPG (umol/L)	5.942 ± 4.38	6.658 ± 4.26

## Data Availability

The data and materials used and analyzed during the current study are available from the corresponding author upon reasonable request.
